# Supplementary material for: Water Use Efficiency and Stress Tolerance of the Potential Energy Crop Miscanthus lutarioriparius Grown on the Loess Plateau of China
Source: Plants (Basel). 2021 Mar 13;10(3):544. doi: 10.3390/plants10030544 (PMC8001145; doi:10.3390/plants10030544)
Supplement: Supplementary file 1 [file plants-10-00544-s001.pdf]

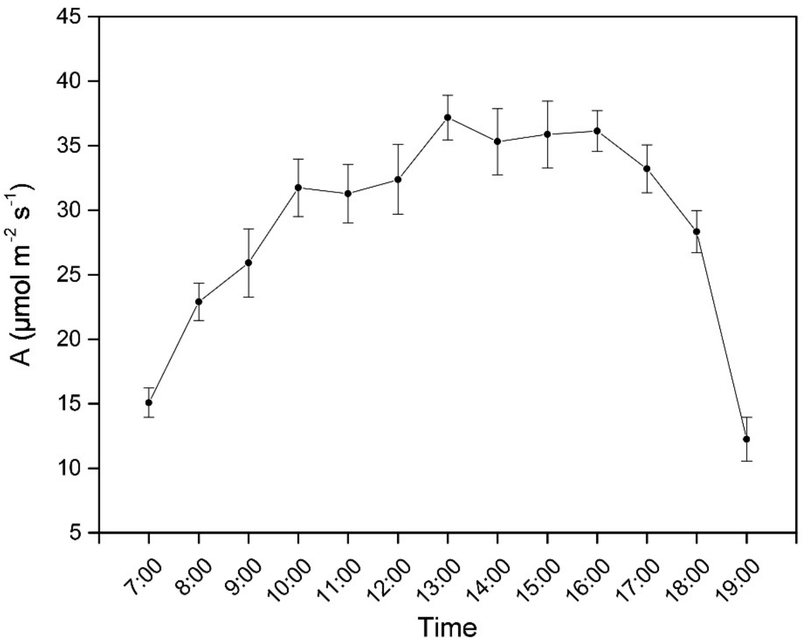

Figure S1. The daily dynamics in photosynthetic rate of *M. lutarioriparius* in HG. The ±SE (error bars) for each time were calculated within the 5 leaves measured repeatedly.

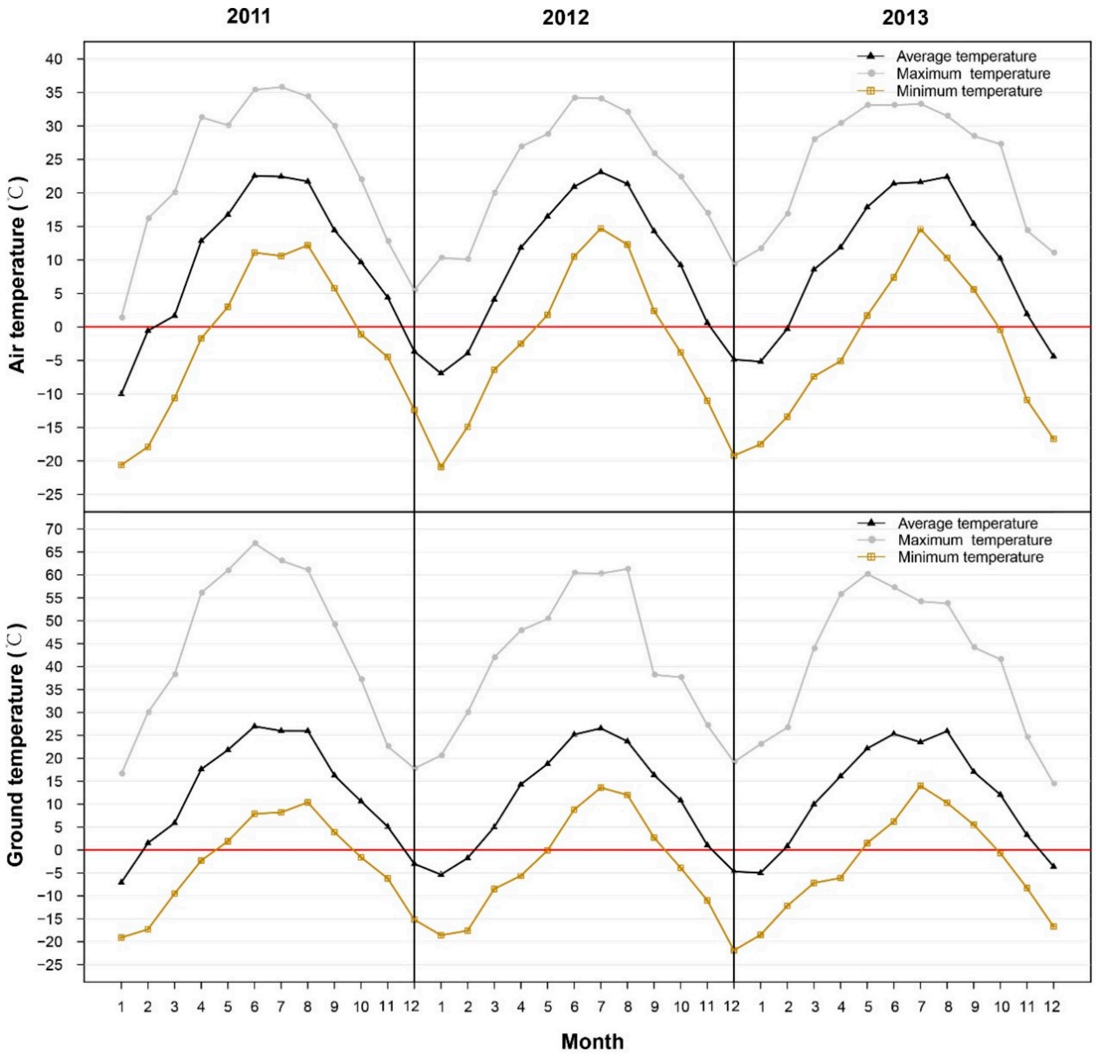

Figure S2. Extreme and average air temperature and ground temperature in HG from 2011 to 2013

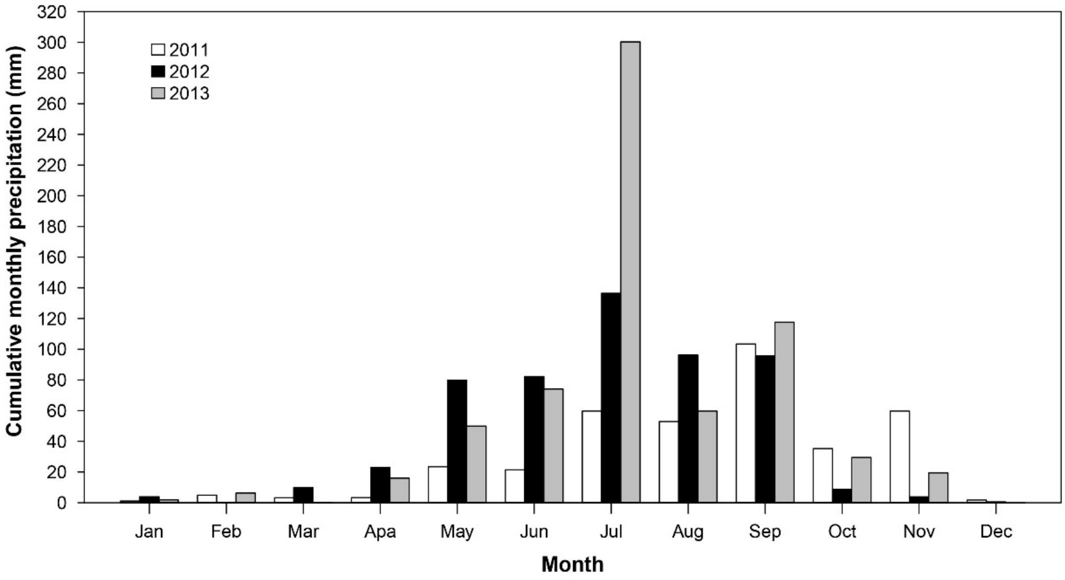

Figure S3. Cumulative monthly precipitation in HG from 2011 to 2013

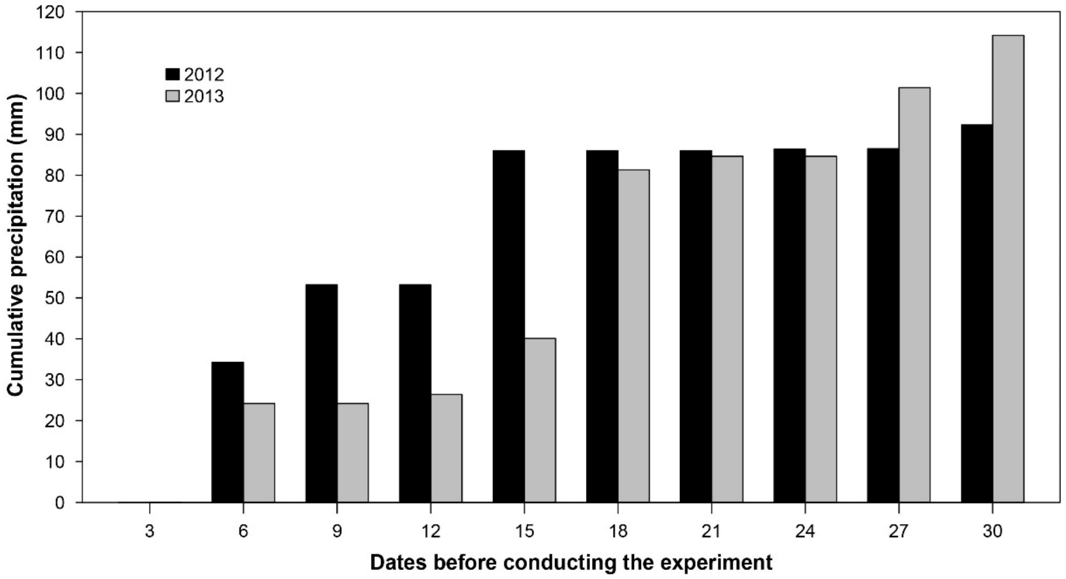

Figure S4. Cumulative precipitation before the measurement of photosynthetic parameters in 2012 and 2013

Table S1. The origin of 41 M. lutarioriparius populations and the times collected

Poppulations	Collection sites	Longitude(E)	Latitude(N)	Collection time
HG01	Yiyang, Hunan	112°19′24.43″	28°37′17.41″	2010
HG02	Xiangying, Hunan	112°53′18.34″	28°48′43.99″	2010
HG03	Yuanjiang, Hunan	112°23′43.78″	28°49′53.45″	2010
HG04	Miluo, Hunan	112°53′47.57″	28°51′57.89″	2010
HG05	Yuanjiang, Hunan	112°19′56.98″	28°59′17.12″	2010
HG06	Nan county, Hunan	112°16′05.89″	29°07′30.69″	2010
HG07	Anxiang, Hunan	112°10′18.66″	29°21′36.15″	2010
HG08	Yueyang, Hunan	113°00′20.38″	29°22′29.39″	2010
HG09	Anxiang, Hunan	112°00′29.95″	29°24′39.17″	2010
HG10	Yueyang, Hunan	113°03′28.82″	29°26′37.43″	2010
HG11	Yueyang, Hunan	112°55′04.95″	29°32′50.48″	2010
HG12	Changde, Hunan	111°52′22.73″	29°33′37.00″	2010
HG13	Honghu, Hunan	113°17′53.52″	29°38′52.04″	2010
HG14	Jingxia, Hubei	114°07′49.78″	30°18′05.77″	2010
HG15	Jiujiang, Jiangxi	116°12′50.55″	29°44′33.85″	2009
HG16	Hukou, Jiangxi	116°09′55.49″	29°44′38.65″	2009
HG17	Jianli, Hubei	112°46′07.96″	29°46′11.93″	2010
HG18	Shishou, Hubei	112°33′32.10″	29°47′46.30″	2010
HG19	Honghu, Hunan	113°29′15.59″	29°47′51.69″	2010
HG20	Jianli, Hubei	112°52′48.55″	29°48′24.85″	2010
HG21	Shishou, Hubei	112°39′56.76″	29°50′50.89″	2010
HG22	Honghu, Hunan	113°34′16.48″	29°51′48.05″	2010
HG23	Jingzhou, Hubei	112°01′48.77″	29°52′56.88″	2010
HG24	Jianli, Hubei	112°55′04.67″	29°58′57.28″	2010
HG25	Pengze county, Jiangxi	116°39′08.05″	29°59′25.88″	2009
HN26	Jiayu, Hubei	113°57′27.01″	30°01′35.06″	2010
HG27	Jiayu, Hubei	114°02′36.30″	30°09′39.49″	2010
HG28	Xiantao, Hubei	113°43′47.49″	30°09′45.95″	2010
HN29	Wuhan, Hubei	113°49′32.89″	30°13′43.03″	2010
HG30	Qianjiang, Hubei	112°47′10.50″	30°25′36.90″	2010
HG31	Wuhan, Hubei	114°10′59.48″	30°27′13.09″	2010
HG32	Anqing, Anhui	117°00′07.34″	30°29′38.40″	2009
HG33	Anqing, Anhui	117°00′40.24″	30°29′55.44″	2009
HG34	Yuanjiang, Hunan	112°24′01.80″	28°49′31.88″	2010
HG35	Wuhu, Anhui	118°00′05.44″	31°07′05.29″	2009
HG36	Wuhu, Anhui	118°17′15.66″	31°14′28.10″	2009
HG37	Anqing, Anhui	120°13′23.19″	31°32′19.00″	2009
HG38	Nanjing, Jiangsu	118°39′39.40″	31°55′43.05″	2009

HG39	Nanjing, Jiangsu	118°49′46.83″	32°09′59.47″	2009
HG40	Zhenjiang, Jiangsu	119°24′44.20″	32°14′35.96″	2009
HG41	Zhenjiang, Jiangsu	119°28′50.18″	32°14′59.27″	2009

Table S2 The annual count of days with extreme temperature d from 2011 to 2013

	Air temperature		Ground temperature	
	2011.10-2012.5	2012.10-2013.5	2011.10-2012.5	2012.10-2013.5
The annual count of days with minimum temperature below 0 degree	132	138	156	155
The annual count of days with average temperature below 0 degree	91	87	81	79
The annual count of days with maximum temperature below 0 degree	28	20	8	3
